# Mycobiome in the Lower Respiratory Tract – A Clinical Perspective

**DOI:** 10.3389/fmicb.2016.02169

**Published:** 2017-01-10

**Authors:** Robert Krause, Christine Moissl-Eichinger, Bettina Halwachs, Gregor Gorkiewicz, Gabriele Berg, Thomas Valentin, Jürgen Prattes, Christoph Högenauer, Ines Zollner-Schwetz

**Affiliations:** ^1^Section of Infectious Diseases and Tropical Medicine, Department of Internal Medicine, Medical University of GrazGraz, Austria; ^2^BioTechMed, Medical University of GrazGraz, Austria; ^3^Institute of Pathology, Medical University of GrazGraz, Austria; ^4^Institute of Environmental Biotechnology, Graz University of TechnologyGraz, Austria; ^5^Division of Gastroenterology and Hepatology, Department of Internal Medicine, Medical University of GrazGraz, Austria

**Keywords:** mycobiome, lower respiratory tract, *Candida*, cystic fibrosis, intensive care unit

## Abstract

Recently the paradigm that the healthy lung is sterile was challenged and it is now believed that the lungs harbor a diverse microbiota also contributing to the pathogenesis of various diseases. Most of the research studies targeting the respiratory microbiome have focused on bacteria and their impact on lung health and lung diseases. Recently, also the mycobiome has gained attention. Lower respiratory tract (LRT) diseases (e.g., cystic fibrosis) and other diseases or conditions (e.g., HIV infection, lung transplantation, and treatment at intensive care units) have been investigated with regard to possible involvement of mycobiome in development or progression of diseases. It has been shown that diversities of mycobiome in the LRT vary in different populations and conditions. It has been proposed that the mycobiome diversity associated with LRT can vary with different stages of diseases. Overall, *Candida* was the dominant fungal genus in LRT samples. In this review, we summarize the recent findings regarding the human LRT mycobiome from a clinical perspective focussing on characterization of investigated patient groups and healthy controls as well as sampling techniques. From these data, clinical implications for further studies or routine practice are drawn. To obtain clinically relevant answers efforts should be enhanced to collect well characterized and described patient groups as well as healthy individuals for comparative data analysis and to apply thorough sampling techniques. We need to proceed with elucidation of the role of mycobiota in healthy LRT and LRT diseases to hopefully improve patient care.

## Introduction

The lower respiratory tract (LRT) includes airways distal of the vocal cords, i.e., trachea, bronchi, bronchioles and anatomical structures of the lung, i.e., respiratory bronchioles, alveolar ducts, alveolar sacs, and alveoli (Schönbach, 2013). Fungal diseases affecting the LRT comprise invasive fungal infections (IFI) and diseases triggered by fungal LRT colonization or inhalation (e.g., allergic bronchopulmonary aspergillosis). Some respiratory diseases are possibly associated with fungal colonization, i.e., exacerbation of chronic obstructive pulmonary disease (COPD) or deterioration of lung function and structure in cystic fibrosis (CF) patients (Nguyen et al., 2015). *Candida* is often cultured from LRT samples but the clinical significance is still unknown. Baum (1960) started a discussion regarding the significance of *Candida* in the LRT by presenting data from conventional microbiological investigations of LRT samples. He described that the source of *Candida* present in the sputum samples was the mouth and that little significance could be attached to the finding of *Candida* within the sputum in the diagnosis of pulmonary candidiasis. Since then subsequent culture based studies investigating *Candida* in the LRT have given conflicting results (Corley et al., 1997; el-Ebiary et al., 1997; Eggimann et al., 2003; Meersseman et al., 2009).

In general, cultivation-independent molecular assays are more efficient in describing microbial communities than culture-based approaches which fail to detect the whole diversity of a microbiota and, therefore, hinder complete understanding of the interactions between the host and the microbiome (Ghannoum et al., 2010; Bittinger et al., 2014; Nguyen et al., 2015). However, compared to bacterial microbiota investigation surveys on the mycobiota of the LRT are still rare. Performing a literature search via PubMed 86 hits are generated by using “mycobiome” as a search term, 9 using “lung mycobiome” and 10 using “respiratory mycobiome” (four of the publications appear also in “lung mycobiome” search^1^; accessed November 21, 2016).

An overview of the current state of knowledge with respect to concepts of LRT mycobiota, the association of LRT mycobiota and fungal communities in other anatomical regions of the human body as well as the role of LRT mycobiota in some LRT diseases has been published recently (Nguyen et al., 2015). In comparison, the LRT mycobiota review presented here primarily addresses the characterization of investigated patient groups and healthy controls as well as utilized sampling techniques. From a clinical point of view, thorough selection of patient groups together with clear description of underlying diseases as well as antiinfective and non-antiinfective treatments that might influence bacterial and fungal microbiota are essential to detect relevant differences in fungal communities. From that informations it might be possible to draw clinically relevant conclusions and diagnostic as well as therapeutic implications. Beside differences in patient selection and molecular approaches, sampling techniques are also relevant for interpretation of results. In this review we therefore summarize recent literature addressing LRT mycobiome and its potential role in respiratory disease in certain patient groups and discuss the results from a clinical perspective including the potential influences by sampling techniques applied.

## Oral Mycobiome

Since LRT samples are commonly collected by procedures wherein the oral cavity is passed, the composition of the oral mycobiota is relevant to interpret LRT mycobiota studies. Although fungal colonization of the oral cavity is not imperatively connected to fungal colonization of other anatomical regions, e.g., the intestinal tract (Zollner-Schwetz et al., 2008), the contact of true LRT samples with saliva and/or mucosal surfaces occurring during expectoration of e.g., sputum samples might alter mycobiota composition in LRT samples. Oral mycobiota were investigated in healthy individuals to provide a basis for a detailed characterization of the oral mycobiome in health and disease. With results of that study the term mycobiome was introduced in medical literature as confirmed by our mycobiome literature search in PubMed starting with Ghannoum’s paper published in 2010 (Ghannoum et al., 2010). In that study, oral rinse using 15 ml of phosphate buffered saline was obtained from 20 healthy subjects (21–60 years of age, eight females and 12 males; non-smoking, no recent antifungal use, and no clinical signs of oral mucosal disease). Exclusion criteria were a history of receiving medication or treatment with topical or systemic steroids, pregnancy, and a history of insulin-dependent diabetes mellitus. Internal transcribed spacer (ITS) based sequencing revealed that the oral cavity contained 74 cultivable (most abundant genera: *Candida*, *Cladosporium*, *Aureobasidium*, *Saccharomycetales*) and 11 non-cultivable fungal genera. The low-abundance genera were considered to represent environmental fungi that have been transferred into the oral cavity by inhalation of spores or by ingesting fungal contaminated food (Ghannoum et al., 2010). As only healthy individuals were investigated the influence of antibacterial and antifungal therapy and certain immunosuppressive conditions predisposing for fungal infections (e.g., underlying diseases like hemato-oncological malignancies, diabetes mellitus, intensive care unit treatment, immunosuppressive treatments like corticosteroids, cyclophosphamide, antineoplastic chemotherapy, biological therapy) on oral mycobiota remain unknown. It should be mentioned, however, that this study lacked appropriate contamination controls and extraction blanks, thus the possible introduction of fungal signatures by the used reagents cannot be excluded.

## Mycobiome of Cystic Fibrosis Patients

In one of the first papers presenting LRT mycobiome data, eight sputum samples from four (two male and two female) adult CF patients (range 19–39 years) were investigated by pyrosequencing of ITS2 amplicons (Delhaes et al., 2012). For assessment of fungal and bacterial interaction 16S rRNA gene sequencing was included. Two temporal sputum samples were collected from each clinically stable patient with a sampling interval of 1 year, but in one patient the sampling interval was only 3 months. Samples were collected by expectoration into a sterile cup after a water rinse. Two patients received azithromycin due to *Pseudomonas* colonization and other unspecified antibiotic therapies (two courses in one patient and up to seven courses in the second patient previous to the first sputum sample). Three patients had inhalative and one patient had additional systemic corticosteroid therapy. One patient was treated with itraconazole 600 mg for 183 days due to bronchopulmonary allergic aspergillosis prior to sampling. Results showed high molecular diversities for the main fungal and bacterial taxa and high intra-individual differences as mycobiota changed between the sampling time points (e.g., 202 *Candida* reads in sample 1 and 8688 *Candida* reads in sample 2 in patient 1). Anaerobes were isolated together with *Pseudomonas aeruginosa*, and the latter was found more likely co-associated with *Candida albicans* than with *Aspergillus fumigatus*. Aside of the bacterial findings the authors concluded that the diversity and species richness of fungal communities was significantly lower in patients with decreased lung function and poor clinical status. It was further mentioned that *C. albicans* and *C. parapsilosis*, as part of the oral mycobiome, could migrate from the oral niche, and colonize and persist within the lower airways of CF patients. However, since *Candida* is the major genus found in the oral cavity as shown in the oral mycobiota study investigating healthy subjects mentioned above and since oral mycobiota have not been investigated in this CF study these conclusions must be interpreted with caution. It is not clear from that CF study whether “sputum” samples represented adequate sputum produced from the LRT and how the oral mycobiota impacted the LRT sputum mycobiota. In addition, the present CF cohort was small (four patients), included CF patients with different treatments that might affect LRT mycobiota (corticosteroids, antibiotics, and antimycotics), and lacked a specific control group. The association of reduced diversity and richness of fungal and bacterial microbiota with poor clinical status and lung function (reduced FEV1 and FVC) was mainly derived from two patients with preceding antibiotic therapy and antifungal therapy. Therefore, influence of antiinfective therapy on reduced microbial diversity and richness cannot be ruled out and the association with lung function remains questionable.

Another study encompassed six CF patients, which were admitted for antibiotic therapy due to respiratory disease exacerbation and *Pseudomonas sp.* positive sputum cultures. ITS based mycobiota analysis was performed with sputum samples collected from this cohort prior to and after systemic antibiotic treatment (Willger et al., 2014). One patient received tobramycin and meropenem; one tobramycin and ceftazidime; one tobramycin, ceftazidime and linezolid; two tobramycin, doripenem and vancomycin; and one tobramycin and doripenem. Inhalative antibacterial therapy (as applied in other CF cohorts as described below) was not mentioned in the studied patients as well as information on preceding antibacterial therapy was lacking. As CF patients repeatedly receive antibacterial (and sometimes antifungal) therapy for infectious exacerbation of underlying disease previous therapies might have impacted the observed LRT mycobiota results. In mycobiota analysis *Candida* represented the dominant genus with 11 different species (top three were *C. albicans*, *C. dubliniensis*, and *C. parapsilosis*) and accounted for 74–99.9% of all ITS1 reads in all samples making these species both the most abundant and also the most prevalent. However, there was a broad distribution of *C. albicans* relative abundance ranging from 0.1% of reads (post-treatment sample from subject number 2) to 98.8% of reads (post-treatment sample from subject number 6). Besides *C. albicans* only *Malassezia species* occurred in all samples with 10- to 50-fold lower reads compared to *Candida species.* Also non-fumigatus *Aspergillus species* were detected at low levels. The relative abundance of *Candida species* was stable during antibiotic treatment and antibiotic treatment did not establish a specific fungal community. Fungal density varied after completion of antibiotic treatment with increase or decrease in individual patients but no common trend could be observed. Overall, no specific trend in fungal burden associated with antibacterial therapy could be detected. In contrast to decreased bacterial richness after antibacterial treatment no significant difference was determined for fungal richness although a trend for a decrease was observed. *Candida* and *Malassezia species* accounted for more than 80% of all assigned reads per sample and persisted throughout antibacterial treatment without significant change in relative abundance suggesting stability within the mycobiome. As with the other CF study, it is not clear if “sputum” samples represented adequate sputum produced from the LRT and if the oral mycobiota contaminated and impacted presumed LRT sputum mycobiota. For further clarification of the role of mycobiota in LRT the authors also suggested methods to differentiate between dead or alive microorganisms to assess active microbiota in the LRT.

In another study 72 sputum samples were collected from 56 CF patients recruited in an ambulatory center including a subcohort of 13 patients who provided sputum two (10 patients) or three (three patients) times within a 2-years sampling period (Kramer et al., 2015a). Sputum samples were collected in duplicate, stored at -20°C and subsequently analyzed by ITS primer based PCR, preparation of single stranded DNA, single-strand conformation polymorphism electrophoresis and sequencing of individual bands. *C. albicans* and *C. dubliniensis* were the most dominant fungal species in the CF cohort, with 44.4% and 23.6% positive samples, respectively, followed by *Saccharomyces cerevisiae*, with 19.4%, and *C. parapsilosis*, with 13.8% positive samples. Several other fungi were detected at lower rates including *Aspergillus sp.*, and *Cladosporium sp.*, *Scedosporium* and *Exophiala sp*. In patients investigated at two time points different fungi were detected in the same patient indicating dynamic mycobiota composition. In concordance with the other CF study mentioned above it is not clear from this particular CF study if “sputum” samples represented adequate sputum produced from the LRT and how oral mycobiota impacted presumed LRT sputum mycobiota (as sputum passes the oral cavity). In another study published by the same group the antibiotic therapy of the same CF cohort was described in more detail. Investigated CF patients were under continuous treatment of antibiotics by inhalation applied twice a day at home (mostly colistin or tobramycin according to clinical needs). Interestingly, none of the patients received antibiotics intravenously during the 2-years time frame of study (Kramer et al., 2015b). Information on antimycotic therapy during or prior to sampling was not provided. Even if the CF cohort represented routine clinical cases conclusions from mycobiota (and microbiota) analysis can hardly be drawn based on the missing information regarding antifungal therapy and the incomplete description of inhalative antibiotic therapy (how many patients received which inhalative antibacterial medication?).

## Mycobiome of Patients with Asthma Bronchiale

Lower respiratory tract mycobiota were investigated in 30 asthma patients and compared to 13 controls (van Woerden et al., 2011, 2013). Patients were free of oral steroid use ≥2 weeks prior to induced sputum sampling but most of them were on inhaled corticosteroids. No information regarding previous or current antibiotic or antifungal therapy was provided. After nebulizing isotonic saline induced sputum was coughed into petri dishes and spread on microscopic slides for microscopic examination. Afterward a 5 mm^2^ area was subsequently excised from the slide for DNA extraction and PCR. The excised samples from each study group were combined to yield two pooled samples, one from all asthma patient sputum samples and one from all healthy control subject sputum samples. Unfortunately, as described by the authors, a sample from one asthma patient was inadvertently included in the control set. Primer pairs Euk1a and Euk516r were used for PCR, sequenced using a 454 pyrosequencer and DNA sequences compared to SILVA database (van Woerden et al., 2013). In total 136 fungal species were identified, with *Psathyrella* and *Malassezia spp.* representing each approximately 25% of all reads in asthma patients and *Eremothecium spp.* representing approximately 40% of all reads in healthy controls. As the authors noted in their discussion pooling sputum samples prevented clear interpretation of results as fungi identified could origin from one individual heavily colonized or from several individuals colonized with identical or nearly similar fungal species. In addition, oral mycobiota as well as material used for sampling (petri dishes, slides, and saline) and its impact on mycobiota results by potential contamination was not investigated. Considering fungal species found in asthma patients the pathophysiological role of *Psathyrella candolleana*, a mushroom found on lawns or pastures in Europe and North America, is unknown as well as the role of *Eremothecium sinecaudum*, a yeast with pathogenic properties for plants, in healthy subjects. The duration for collection of pooled sputum and time intervals between sample collection and storage was not provided as well as storing conditions. It has been shown that prolonged time between sample collection and sample storage (more than 12 h) as well as more than four freeze thaw cycles distorted microbiota profiles (Cuthbertson et al., 2014, 2015). As mentioned above, fungi found in sputum from asthma patients and healthy controls mainly contained environmental fungi present in plants and grassland (van Woerden et al., 2013). Thus contamination of the pooled sputum from both study groups during sampling and storage cannot be ruled out. As sputum samples were mixed up from individual patients and the pooled control sample included one asthma sample interpretation of results and considerations of differences between study populations is very limited.

## Mycobiome of Patients with Lung Transplants

Mycobiota based on ITS-sequencing of 21 lung transplant patients were investigated in BAL and oropharyngeal washes and compared to healthy controls, the latter undergoing a two-bronchoscope sampling technique or conventional bronchoscopy (number of healthy controls undergoing each of the sampling techniques was not provided in the text; in figures within the manuscript data from six healthy controls were shown). Two patients undergoing routine bronchoscopy for evaluation of sarcoidosis and a lung nodule representing adenocarcinoma (but both without lung transplantation) were also included, but they were not further characterized (Charlson et al., 2012). All lung transplant patients had immunosuppressive and antibiotic therapy, three received voriconazole and six nystatin (one patient had both antifungals). Prior to bronchoscopy instruments were flushed with 10 ml saline to investigate microbial DNA present in bronchoscopes and saline. Fungal populations in lung transplant patients were typically dominated by *Candida* in oral washes and BAL and by *Aspergillus* in BAL but not in oral washes. Oral washes of lung transplant patients revealed 13 samples with high fungal sequences, all but one were dominated by *Candida*. Patients without *Candida* in BAL had no (six patients) or low (two patients) *Candida* sequences in oral washes. As eight lung transplant patients had nystatin and/or voriconazole therapy the presence or absence of *Candida* and other fungi (targeted by nystatin and voriconazole) in oral washes and BAL is hard to assess. Nystatin did not consistently decrease or eliminate *Candida* from oral washes but patients on voriconazole had no *Candida* sequences in BAL (patient no. 34 was only presented in Figure E2, supplementary material of the article; Charlson et al., 2012). However, in two of voriconazole treated patients *Aspergillus* sequences were detected (patient no. 34, also with positive *Aspergillus* culture, and patient no. 43). One patient (no. 36) had *Aspergillus* in culture and in mycobiota analysis but in a second BAL 2 months later *Aspergillus* was no longer detected by both methods while the patient was treated with voriconazole; voriconazole obviously eliminated *Aspergillus* in that particular patient. *Candida* was absent in BAL of healthy volunteers although *Candida* was present in oral washes. Unfortunately no information regarding a potential pathogenetic role of fungi detected within mycobiota was provided for the presented lung transplant patients. The presence or absence of invasive fungal infection or fungal breakthrough infection in voriconazole treated patients (based on clinical data, radiological examination, biomarkers like galactomannan or 1,3-beta-D-glucan testing, voriconazole trough level) and outcome was not mentioned.

## Mycobiota in Miscellaneous Pulmonary Diseases and HIV Positive Patients

In a study published by the same authors as mentioned above in the lung transplant patients, data from an almost similar study cohort included mycobiota from 42 lung transplant patients, 19 HIV positive patients, 13 patients with various pulmonary diseases and 12 healthy controls (Bittinger et al., 2014). Four control patients were counted twice as they were investigated by a double bronchoscopy and single bronchoscopy technique at least 1 year apart. Healthy and HIV positive subjects were described not to have respiratory symptoms and antibiotic therapy. However, in the **Table 1** one HIV patient did in fact have antibiotic therapy (clindamycin within 3 months prior to sampling) and another one had inhalative corticosteroid treatment. Subjects within the healthy and HIV positive study groups where smokers and non-smokers. In patients with various pulmonary diseases two patients had immunosuppressive therapy, two had systemic antibiotic therapy but two patients with pneumonia surprisingly had no antibiotic therapy. Two out of 24 lung transplant patients refused collection of clinical data. Thirteen of the remaining 22 lung transplant patients had antifungal therapy (11 nystatin, two voriconazole treatments). Twenty received antibiotic therapy (mainly trimethoprim and sulfamethoxazole), two had atovaquone therapy and all received immunosuppressants (presented in Supplementary Table S3 of Bittinger et al., 2014). The authors also focused on analysis of possible confounding factors as 132 samples from laboratory water, bronchoscope canal flushes, saline used for oral washes and BAL, sterile swabs and lab tabletop surface were investigated [supplemental report in (Bittinger et al., 2014)]. Interestingly, the composition of fungi in BAL samples was generally similar to that in contamination controls. After correction of abundance by subtraction of reads found in the control samples (e.g., saline) *Candida* was mainly found in BAL and oral washes of the study groups (Figure 37–43 in supplemental report of Bittinger et al., 2014) as well as *Aspergillus* and *Cladosporium* in lung transplant patients and those with miscellaneous pulmonary diseases (Figure 37 and 38 in supplementary report of Bittinger et al., 2014). Clinical significance of fungal detection in presented patient groups is unclear from that study as clinical data including radiological examination, biomarkers, introduction of antifungal treatment and outcome have not been reported.

**Table 1:** Overview of studies investigating LRT mycobiota.

Study author, year of publication	Patients (pts)/study subjects (controls)	Sampling method	Material analyzed
Delhaes et al., 2012	four CF pts	sputum expectoration	sputum
Willger et al., 2014	six CF pts	sputum expectoration	sputum
Kramer et al., 2015a	56 CF pts	sputum expectoration	sputum
van Woerden et al., 2013 van Woerden et al., 2011	30 asthma bronchiale pts 13 controls	induced sputum expectoration	two pooled sputum samples (one from patients and one from controls)
Charlson et al., 2012	21 lung transplant pts one patient with suspected sarcoidosis one patient with adenocarcinoma	bronchoscopy (including 2-bronchoscope sampling in healthy controls) oral washes with saline bronchoscope saline flush	BAL Oral washes saline and bronchoscope flush
	six controls		
Bittinger et al., 2014	42 lung transplant pts 19 HIV pts 13 pts with various pulmonary diseases 12 controls	bronchoscopy (including 2-bronchoscope sampling in healthy controls) oral washes with saline bronchoscope saline flush swabs	BAL oral washes laboratory water bronchoscope flushes saline sterile swabs lab tabletop surface
Cui et al., 2015	32 HIV pts [10 with chronic obstructive pulmonary disease (COPD)] 24 HIV negative pts	oral washes with saline induced sputum bronchoscopy	oral washes sputum BAL
Bousbia et al., 2012	160 ICU pts with pneumonia 25 ICU pts without pneumonia	bronchoscopy	BAL
Krause et al., 2016	39 ICU pts seven non-ICU patients with antibiotic therapy five ICU pts for sampling comparison eight controls	bronchoscopy through endotracheal tubes suction of endobronchial secretion through endotracheal tubes bronchoscope saline flush	BAL endobronchial secretion bronchoscope flushes saline


Another study provided mycobiota data from 32 HIV positive (10 with COPD) and 24 HIV-uninfected patients extracted from a cohort comprising 396 HIV-infected and HIV-uninfected individuals with smokers and marijuana abusers in both study groups (Cui et al., 2015). Prior to sampling study subjects had stable respiratory symptoms in previous 4 weeks and did not receive antibiotic or immunosuppressive therapy in previous 6 months. Oral wash, induced sputum (<30% squamous cell count) and BAL passing the oral cavity were collected as well as control samples by aspirating saline through the bronchoscope channel. Fungal microbiota were analyzed by sequencing amplicons generated by PCR using ITS- and 18S- targeting primers. 18S data were used for operational taxonomic unit (OTU)-type analysis and ITS data were used for species-level analysis. The authors mentioned that the 18S rRNA gene gave higher PCR-positive rates, but fewer fungal reads compared with the ITS, because the 18S primers cross-reacted with host (=human); consequently, approximately 90% of the raw 18S sequencing reads were human. By using 18S data for comparison of different sampling techniques, the authors interestingly found that induced sputum shared little similarity with BAL. In principle coordinate analysis of 18S data of healthy subjects (HIV negative, normal lung function) using only 30% of all data, BAL samples clustered together with oral wash, induced sputum overlapped in part with oral wash samples but not with BAL. A similar distribution pattern was seen with the entire cohort including HIV-infected and HIV-uninfected individuals with or without normal lung function. *Candida* represented more than 90% of the ITS reads and was clearly dominant in oral washes compared with BAL. There was also a higher prevalence of *Candida* in induced sputum compared with BAL. *Ceriporia lacerata*, *Saccharomyces cerevisiae*, and *Penicillium brevicompactum* were predominant in BAL compared to oral wash. None of these species were detected in the corresponding bronchoscopic control samples, indicating that environmental contamination was unlikely. In HIV infected individuals nine species including *Pneumocystis jirovecii* (confirmed by nested PCR) were overrepresented in BAL compared to HIV-uninfected individuals. No species was associated with COPD in the HIV-infected cohort as compared with HIV-infected individuals with normal lung function. In marijuana smokers, with and without HIV infection, four species (including two that are known plant pathogens, *Phialocephala virens* and *Taphrina tormentillae*) were overrepresented as compared with individuals who had not smoked marijuana in the year prior to sampling. The authors mentioned that smoking marijuana without filters might have contributed to inhalation of this certain environmental/plant fungi. Considering all subjects investigated, fungal communities in the LRT resembled those in the mouth. This result could be caused by microaspiration of oral content, contamination during bronchoscopy when passing the oral cavity, contamination of induced sputum with oral microbiota or contamination of oral microbiota by coughing or exhalation. The latter is unlikely as oral wash was collected prior to sputum sampling or bronchoscopy.

## Mycobiome in LRT of ICU Patients

Mycobiota in LRT of ICU patients were investigated in two studies. In the first study 18S rRNA gene sequencing was performed to analyze the etiology of pneumonia in ICU patients with community acquired pneumonia (CAP, 32 episodes), ventilator-associated pneumonia (VAP, 106 episodes), non-ventilator ICU pneumonia (NV-ICU P, 22 episodes), aspiration pneumonia (AP, 25 episodes) and compared to 25 ICU patients without pneumonia (CS; Bousbia et al., 2012). As pneumonia cases were counted as episodes (185 pneumonia episodes) the total number of patients included in each of the pneumonia subgroups is not clear. However, in some tables the number of pneumonia patients were 185 enabling the conclusion that one pneumonia episode obviously corresponded to one patient. Clinical data regarding underlying diseases beside pneumonia, (previous) antibiotic and antimycotic therapy or initiation of new antiinfective therapy, presence or absence of tracheal tubes, invasive or non-invasive mechanical ventilation and underlying diseases were not provided. Detailed sampling procedures, e.g., time of BAL sampling after admission or diagnosis or pneumonia, BAL by bronchoscopy passing the oral cavity or through oropharyngeal ventilation tubes, transport and storage of BAL cultures, investigation of material used for BAL, investigation of pre-BAL bronchoscopy flushes were not mentioned. Interestingly, ICU patients used as control patients (=no pneumonia) also had ARDS but with comparable or even higher numbers compared to some pneumonia patients (38% in CAP, 33% in VAP, 41% in NV-ICU P, 12% in AP; 31% in all pneumonia patients and 28% in patients without pneumonia considered as controls). The proportion of patients with immunosuppressive therapy in each pneumonia group was reported but the type of immunosuppressive therapy was not provided (38% in CAP, 40% in VAP, 50% in NV-ICU P, 36% in AP; 40% in all pneumonia patients and 16% in controls). *C. albicans* signatures were most abundant in all patients groups and controls. *C. utilis* showed higher abundance in controls compared to pneumonia subgroups (*p* = 0.01; Supplementary Table S5 in supplementary material of Bousbia et al., 2012). *Aspergillus*, *Penicillium* and *Cladophialophora* genera were dominant in the CAP cohort but did not reach significant differences compared to other cohorts. *Tremellomycetes* (represented by *Cryptococcus*) was identified in NV-ICU P, whereas *Agaricomycetes* and unclassified *Ascomycota* were only identified in VAP patients. *Sordariomycetes* were only identified in controls. However, maybe based on small numbers, no significant difference could be calculated for these classes. Beside bacterial and fungal microbiome analysis the authors included a lot of other microbiological methods to detect microorganisms causing pneumonia. For many of detected microorganisms including those found in mycobiota analysis the authors raised the question on the actual role of these microorganisms in pneumonia. The authors concluded that the respiratory microbiota was more complex than expected (Bousbia et al., 2012).

In another study, LRT mycobiota in non-neutropenic intubated and mechanically ventilated ICU patients with antibiotic therapy to treat pneumonia were investigated and compared to several other groups: healthy controls without preceding antibiotic therapy, patients without pulmonary diseases with antibiotic therapy, non-neutropenic intubated and mechanically ventilated ICU patients without preceding or current antibiotic therapy, non-neutropenic intubated and mechanically ventilated ICU patients without pulmonary disease but with antibiotic therapy for treatment of extrapulmonary infection. Smoking behavior of study patients was not evaluated. All study subjects were without antimycotic treatment ≥8 weeks prior to sampling (Krause et al., 2016). LRT in all patients and healthy study subjects were sampled through endotracheal tubes avoiding contact to the oral cavity. Non-ICU patients and healthy controls were sampled by aspiration of endobronchial secretion and ICU patients were sampled by bronchoscopic guided BAL. Saline used for BAL and collected after flushing the sampling channel of the bronchoscope immediately before bronchoscopy was also investigated. Sampling techniques (endobronchial secretion versus BAL) showed similar results and saline did not influence mycobiota results. The results showed that *Candida* was part of the fungal microbiota of various intubated and mechanically ventilated ICU patients with and without antibiotic therapy and with and without pneumonia (59–73% *Candida* signatures within 5–8 fungal genera; Krause et al., 2016). *Candida* sequences were not present in mycobiota of non-ICU patients with antibiotic therapy and healthy controls. Admission to and treatment on ICUs shifted LRT fungal microbiota to *Candida* spp. dominated fungal profiles but antibiotic therapy did not. Mycobiota analysis was not done with samples from the oral cavity but in conventional cultures antibiotic therapy increased the prevalence of *Candida* spp. in this compartment. Conventional cultures also showed that admission to the ICU increased the prevalence of *Candida* spp. in the LRT, but other fungi were not detected by culture. None of the ICU patients had candidemia or other invasive fungal disease as determined by clinical, imaging, laboratory and microbiological investigations.

## Discussion

The mycobiota in the LRT is diverse and varies in different populations and LRT diseases. Links to certain stages of diseases or LRT dysfunction have been proposed. In one study the diversity and species richness of fungal and bacterial communities were significantly lower in patients with decreased lung function as assessed by forced expiratory pressure in 1 s in lung function tests (Delhaes et al., 2012). However, the association was mainly derived from two patients with preceding antibiotic therapy and antifungal therapy and the contribution of fungal or bacterial microbiota to lung function could not be distinguished. Despite variable fungal composition in LRT, *Candida* overall dominated mycobiota in most of the studies including CF patients, lung transplant patients, HIV infected patients and ICU patients (Bousbia et al., 2012; Charlson et al., 2012; Bittinger et al., 2014; Willger et al., 2014; Cui et al., 2015; Kramer et al., 2015a; Krause et al., 2016). In remaining studies with dominating fungi other than *Candida* methodologies were too weak to draw profound conclusions. However, *Candida* was also the most abundant genus in oral mycobiome analysis (Ghannoum et al., 2010) questioning the results of studies that presented sputum samples without quality control (e.g., <10 squamous cells in high power field sputum examination). Unfortunately, even in bronchoscopy guided BAL contact to oral mucosa or saliva during insertion of the bronchoscope might alter microbiota composition of BAL obtained through orally contaminated bronchoscopy tips. But LRT mycobiota similar or close to oral mycobiota does not necessarily represent contamination of bronchoscopes or translocation of mycobiota by bronchoscopy, as upper and LRT might be colonized by identical or closely related microorganisms. Similar or close mycobiota in upper and LRT analysis could also be caused by microaspiration of oral content, contamination of induced sputum with oral microbiota or contamination of oral microbiota by coughing or exhalation. To overcome limitations in interpreting mycobiota data sampling techniques bypassing the oral cavity (e.g., via endotracheal tubes) or two-bronchoscopy sampling techniques should therefore be preferred to obtain true LRT samples. By applying such techniques and therefore exclusion of contamination, mycobiota in upper and LRT could be assessed more reliable.

In two previous studies reagents (e.g., saline, lab water, lab surfaces) have been analyzed to assess its influence on microbiota results from BAL (Bittinger et al., 2014; Krause et al., 2016); however, in other studies this was not done (Erb-Downward et al., 2011; van Woerden et al., 2011, 2013). Nevertheless, none of the studies investigating reagents showed that *Candida* abundance reached >50% as has been found in BAL samples from ICU patients (Krause et al., 2016). The reason for the elevated *Candida* abundance in LRT of ICU patients is not clear. The role of ICU environment or translocation of microorganisms from intestinal mycobiota (via ICU personnel or retrograde translocation up to the oral cavity followed by microaspiration passing the cuff or the orotracheal tube) in elevated LRT *Candida* abundance or in shifts of LRT mycobiota has not been investigated yet.

Three studies included sequential sampling for assessment of dynamics in mycobiota composition over time. Observation periods varied between days (ICU patients; Krause et al., 2016), weeks (CF patients; Willger et al., 2014), 3 months to 1 year (CF patients; Delhaes et al., 2012), and months up to 2 years (CF patients; Kramer et al., 2015a). Conclusions are difficult to draw based on low numbers of individuals investigated, different patient groups and differences in time frames elapsing between sampling. Whereas high intraindividual differences with changes in *Candida* abundance were found in one CF study (Delhaes et al., 2012) another CF study found stable results (Willger et al., 2014). The third CF study found differences in number of fungal genera in some patients providing two sputum samples (Kramer et al., 2015a). In ICU patients fungal microbiota were stable in 3 of 4 patients as shown by analysis of first and second BAL sample 4 to 7 days apart (Krause et al., 2016). Two studies compared different LRT sampling techniques in individual patients, i.e., induced sputum and BAL in one study (Cui et al., 2015) and endotracheal aspiration and BAL in another study (Krause et al., 2016). Whereas differences were detected between sputum and BAL based on 18S rRNA sequencing no difference was found between endotracheal aspirate and BAL based on ITS sequencing. As the authors mentioned in 18S based sequencing human 18S rRNA reads reduced the sequencing depth of fungal 18S rRNA gene reads, which might have influenced comparative data analysis of sputum and BAL. Although some studies included conventional cultures for assessment of microbial composition all studies mentioned above did not account for viability of microbiota explored by sequencing methods. Therefore it is almost unknown if detected mycobiota represent true alive residential or inhaled dead microorganisms or both. Future studies need to include this aspect by e.g., dividing samples and treating of one half with propidium monoazide (PMA) to select for dead cells (Nocker et al., 2007).

The role of *Candida* in LRT in above mentioned diseases and conditions is still largely unknown. Patient characteristics provided in some mycobiota studies prohibit clinical conclusions as relevant data regarding underlying diseases, presence or absence of LRT infectious disease, initiation of antibiotic or antimycotic therapy, and outcome (e.g., invasive fungal infection) are missing. In the study investigating asthma bronchiale patients no information regarding previous or current antibiotic or antifungal therapy was provided (van Woerden et al., 2013). As shown in one of the studies in ICU patients (Krause et al., 2016) antibiotic therapy surprisingly did not influence mycobiota. Interestingly, antibiotic therapy in CF patients overall also had no impact on mycobiota composition and fungal burden although fungal burden varied between individual patients with decreasing and increasing levels (Willger et al., 2014). The influence of antimycotic therapy on LRT mycobiota is still unclear but some antimycotics might alter LRT mycobiota as detected in one patient in the lung transplant patient group discussed above (Charlson et al., 2012).

## Conclusion

Although some studies suggest renewal of our pathophysiological understanding of mycobiota in LRT, shortcomings in clinically relevant methodologies in some studies call for further investigations to compensate previous deficiencies. Improved study settings will allow to draw better clinically relevant conclusions regarding diagnostic or therapeutic procedures in certain LRT diseases. As previously suggested, future mycobiota (and bacterial microbiota) studies should enable to answer one or more of the following questions: Can experiments detect differences that matter (in the context of disease and treatment)? Does the study show causation or just correlation? What is the mechanism? How much do experiments reflect reality? Could anything else explain the results? (Hanage, 2014). To obtain clinically relevant answers efforts should be enhanced to collect well characterized and described patient groups as well as healthy individuals for comparative data analysis and to apply thorough sampling techniques. For clinical routine we learn from current stage of knowledge that conventional microbiology obviously does not reflect true composition of LRT mycobiome. However, therapeutic studies based on new sequencing techniques are missing. We need to proceed with elucidation of the role of mycobiota in healthy LRT and LRT diseases to hopefully improve patient care. This might include diagnostic procedures with cultivation-independent molecular assays and prophylactic as well as therapeutic interventions in patients potentially suffering from mycobiota alterations (e.g., CF patients, lung transplant patients, ICU patients).

## Author Contributions

All authors listed, have made substantial, direct and intellectual contribution to the work, and approved it for publication.

## Conflict of Interest Statement

The authors declare that the research was conducted in the absence of any commercial or financial relationships that could be construed as a potential conflict of interest.

## Footnotes

1 http://www.ncbi.nlm.nih.gov/pubmed/?term=mycobiome
